# A New Method for Morphometric Analysis of Tissue Distribution of Mobile Cells in Relation to Immobile Tissue Structures

**DOI:** 10.1371/journal.pone.0015086

**Published:** 2011-03-08

**Authors:** Liudmila Nikitina, Helmut Ahammer, Astrid Blaschitz, Angela Gismondi, Andreas Glasner, Michael G. Schimek, Gottfried Dohr, Peter Sedlmayr

**Affiliations:** 1 Institute of Cell Biology, Histology and Embryology, Medical University of Graz, Graz, Austria; 2 Institute of Biophysics, Medical University of Graz, Graz, Austria; 3 Department of Experimental Medicine, University of Rome “La Sapienza”, Rome, Italy; 4 Gynecological practice, Seiersberg, Austria; 5 Institute for Medical Informatics, Statistics and Documentation, Medical University of Graz, Graz, Austria; Health Canada, CANADA

## Abstract

The distribution of cells in stained tissue sections provides information that may be analyzed by means of morphometric computation. We developed an algorithm for automated analysis for the purpose of answering questions pertaining to the relative densities of wandering cells in the vicinity of comparatively immobile tissue structures such as vessels or tumors. As an example, we present the analysis of distribution of CD56-positive cells and of CXCR3-positive cells (relative densities of peri-vascular versus non-vascular cell populations) in relation to the endothelium of capillaries and venules of human parietal decidua tissue of first trimester pregnancy. In addition, the distibution of CD56-positive cells (mostly uterine NK cells) in relation to spiral arteries is analyzed. The image analysis is based on microphotographs of two-color immunohistological stainings. Discrete distances (10–50 µm) from the fixed structures were chosen for the purpose of definining the extent of neighborhood areas. For the sake of better comparison of cell distributions at different overall cell densities a model of random distribution of “cells” in relation to neighborhood areas and rest decidua of a specific sample was built. In the chosen instances, we found increased perivascular density of CD56-positive cells and of CXCR3-positive cells. In contrast, no accumulation of CD56-positive cells was found in the neighborhood of spiral arteries.

## Introduction

In many instances it is of interest to analyze the tissue distribution of mobile cells in relation to comparatively immobile tissue structures. This may be the case for analysis of the result of extravasation of cells of the immune system from the blood into the tissue, where cell populations bearing a receptor involved in the extravasation process, having passed through the vessel wall are preferentially located in the vicinity of the vessel before this receptor may be downregulated. Further examples include analysis of the result of cell migration to certain structures in the tissue, such as surface epithelia, tumors or artificial implants. Analysis of the tissue distribution of specific cell types may complement *in vitro* assays of cell migration.

We developed a method for morphometric analysis of tissue distribution of mobile cells in relation to immobile tissue structures and used this methodology to address three different questions.

(1) Analysis of the distribution of CD56^+^ cells in the decidua in relation to capillaries and venules (vessels devoid of a tunica media composed of smooth muscle cells).

The main component of CD56^+^ cells in the decidua is made up by a specific subset of Natural Killer (NK) cells which constitutes the main leukocyte population of the human decidua during the first trimester of pregnancy [1]. This population of uterine NK (uNK) is characterized by a CD16^-^, CD56^bright+^ phenotype and low lytic activity [2]. It was suggested that uNK cells are involved in the processes of trophoblast invasion and vascular remodeling of spiral arteries, this way increasing the blood flow to the feto-maternal interface [3]. They may do so by means of their array of products such as cytokines, chemokines and angiogenic factors [4], [5]. Our method should be able to tell us whether uNK cells are relatively enriched in the vicinity of capillaries and venules which are sites of extravasation of leukocytes from blood into the decidua tissue [6] .

(2) Analysis of the distribution of CXCR3^+^ cells in relation to capillaries and venules (vessels devoid of a tunica media composed of smooth muscle cells).

Compared with the chemokine receptor pattern of peripheral blood NK cells, it is particularly the chemokine receptor CXCR3 which is expressed at a higher degree by uNK cells whereas others are at lower or undetectable levels, which is consistent with the known profile of uNK cells for migratory response [7], [8]. However, the question is not yet fully resolved to what extent proliferation of residual uNK cells and migration and differentiation of peripheral blood NK cells or bone marrow precursors into the uterus contribute to the accumlation of uNK cells during early pregnancy [6], [9]. Both the small CD56^bright+^ blood NK cell population and uNK cells express this receptor for the ligands CXCL10 and CXCL11 which are produced by the human endometrium under the influence of estrogen [10]. CXCR3 expression in the decidua is not exclusively restricted to NK cells, however, as it is found also on the comparatively small population of T cells (data not shown). We applied our method to determine whether CXCR3^+^ cells relatively enriched in the vicinity of capillaries and venules, which would contribute to evidence that this chemokine receptor is involved in the recruitment of peripheral blood human NK cells to the decidua.

(3) Analysis of the distribution of human CD56^+^ cells in relation spiral arteries of the decidua.

Data from the mouse suggest dependence of vascular remodeling in the decidua (transformation of spiral arteries containing a tunica media composed of smooth muscle cells to wide flaccid vessels devoid of smooth musculature) on uNK cell derived INFγ [11]. This prompted us to apply our method for analysis whether human uNK cells are relatively enriched in the vicinity of the tunica media of spiral arteries in first trimester decidua.

**Figure 1:**
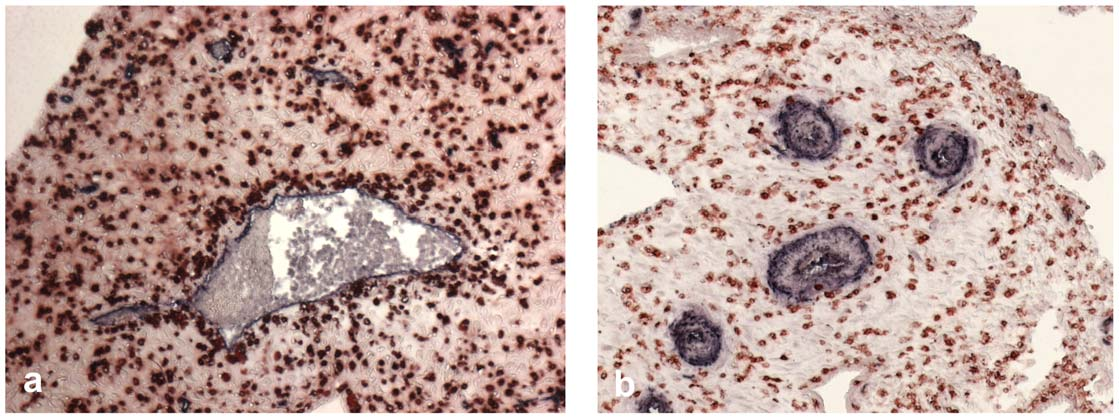
Examples of microphotographs for automated analysis: Two-color immunohistochemistry of non-invaded first trimester decidua. (a) Red: (mostly uterine NK) cells stained with anti-CD56-HRP-AEC, blue: CD34^+^ vascular endothelium stained with anti-CD34-APAAP-NBT/BCIP. (b) Red: CD56^+^ cells, blue: tunica media of spiral arteries stained with anti-smooth muscle actin – APAAP-BCIP.

**Figure 2:**
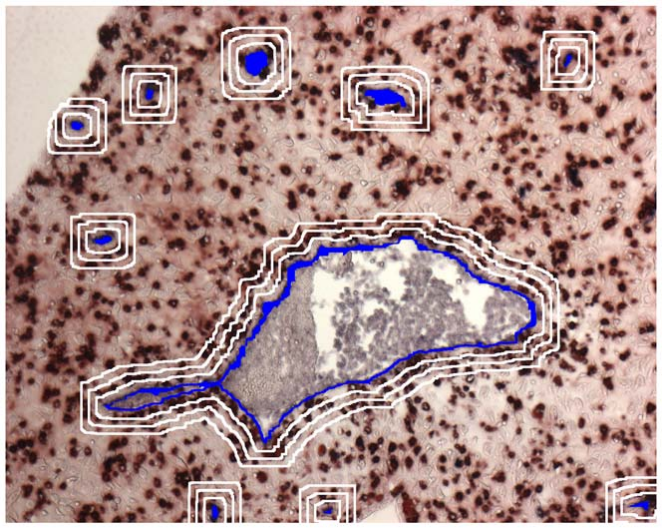
Definition of neighborhood area on the basis of a microphotograph of two-color immunohistochemistry of first trimester decidua (see Figure 1a). Generation of an area of vicinity of a size to be chosen (in our example, 3 sizes at distances of up to 10, 20, and 30 µm from the vascular endothelium are given).

Our analysis is based on tissue sections stained with 2-color immunohistochemisty, where one color characterizes the mobile cells to be examined and the other one the fixed structures (Examples in Figure 1). In the course of the analysis areas of neighborhood of the fixed structures are defined and the density of the mobile cells inside the neighborhood is compared to the residual tissue outside the neighborhood area (for illustration of neighborhood areas see Figure 2).

## Materials and Methods

### Ethics

The study was approved by the Ethics Committee of the Medical University of Graz (No. 16–188 ex 04/05). The Ethics Committee of the Medical University of Graz (Ethikkommission der Medizinischen Universität Graz, http://www.meduni-graz.at/ethikkommission/Graz/) is registered at the Office for Human Research Protections (OHRP) of the US Department of Health and Human Services (DHHS) with the number IRB00002556. Written informed consent was obtained from all participants involved in the study.

### Tissue material

31 samples of first trimester decidual tissue (6^th^–11^th^ week of pregnancy) were obtained from elective pregnancy terminations by curettage and vacuum suction and resulted in tissue material with sufficient quality for ensuing immunohistological preparation. In tissue from 15 patients the distribution of CD56^+^ cells and in tissue from 17 patients the distribution of CXCR3^+^ cells was tested in relation to endothelium of vessels devoid of a layer of smooth musculature, in tissue samples from 14 patients analysis of the distribution of CD56^+^ cells was done in relation to the ring of smooth muscle cells in the tunica media of spiral arteries. From individual patients between one and five tissue samples were available for analysis.

In order to exclude skewing of the tissue distribution of CD56^+^ cells and of CXCR3^+^ cells by the presence of invading extravillous trophoblast, samples of decidua basalis were excluded from further analysis. Trophoblast invasion was excluded by immunohistochemistry based on cytokeratin 7 staining or by morphological evaluation.

Samples of decidua tissue (from 16 patients) which were later used for analysis of distribution of CD56^+^ cells were fixed using the HOPE (HEPES-glutamic acid buffer mediated organic solvent protection effect) method [12]. The tissue was consecutively treated with HOPE I solution (CDS Diagnostics, Hamburg, Germany) for 16–72 h, then with HOPE II solution diluted in acetone (1∶1000) for 2 h and afterwards dehydrated three times with pure acetone for 2 h each. All incubation steps for HOPE-fixation were carried out at 0–2°C. Specimens were embedded in low temperature paraffin, cut into 5 µm slices and mounted on SuperFrost Plus slides (Menzel-Gläser, Brauchschweig, Germany).

For analysis of distribution of CXCR3^+^ cells, we used samples of tissues from 17 patients. Samples from 16 patients were fixed in 4% paraformaldehyde (PFA) for at least 5 d and paraffin-embedded. In addition, HOPE-fixed and paraffin-embedded samples from one patient were used.

### Antibodies

The primary monoclonal antibody anti-human-CD183 (CXCR3) (mouse IgG1 isotype, clone 1C6/CXCR3, Becton-Dickinson, Schwechat, Austria) was used at a concentration of 5 µg/mL. Mouse anti-human CD56 (IgG1, Immunotech, Marseille, France) was used at a dilution of 1∶200, anti-human CD34 (mouse IgG1, clone QBEND10, Dako) at 0.05 µg/mL. For negative controls mouse IgG1 (Dako, Glostrup, Denmark) was used at corresponding concentrations. The second step antibodies (goat anti-mouse-Ig-biotin and rabbit anti-mouse-HRP-Polymer) were provided in the detection kits (see below). For exclusion of trophoblast infiltration anti-cytokeratin 7 was used (clone OV-TL 12/30, Thermo Scientific, Eubio, Vienna, Austria).

### Immunohistochemistry

The sections were deparaffinized in xylene (2×10 min) followed by short washes in 100%, 96% and 70% ethanol. All slides were rinsed in distilled water. Slides from PFA-fixed specimens only were treated in a decloaking chamber (Biocare, Sanova, Vienna) at 120 °C at pH 9 for 7 min. The slides were then rehydrated in TBS-T (tris-buffered saline supplemented with 0.05% Tween 20, pH 7.2), a hydogen peroxide block (Lab Vision, Fremont, CA) was used. Incubation was done with UV block (Lab Vision; which was supplemented with 10% human AB serum in case of HOPE-fixed specimens) followed by primary antibodies (anti-CXCR3 or anti-CD56) for 40 min at room temperature, the slides were rinsed thoroughly in TBS and exposed to the labeled polymer horseradish-peroxidase (HRP) system (Lab Vision) following the manufacturer's instructions. The peroxidase was developed with 3-amino-9-ethyl-carbazole/dimethylformamide (AEC) in acetate buffer in the dark for 10 min. The reaction was stopped by rinsing in distilled water. For the second labeling step the UltraVision Mouse Tissue Detection System Anti-Mouse, Alk-Phos/BCIP/NBT (Lab Vision) was used. For this purpose the slides were blocked with UV block for 3 min and incubated for 40 min with the second primary antibody (anti-CD34 od anti-SMA) at room temperature. The sections were then washed in TBS, incubated with goat anti-mouse biotinylated antibody for 10 min and then with streptavidin alkaline phosphatase for 15 min, rinsed thoroughly in TBS and developed with the chromogen NBT/BCIP (nitro blue tetrazolium chloride/5-bromo-4-chloro-3-indolyl phosphate, toluidine salt, Lab Vision) for 10 min in the dark. Using single color staining with both the first and the second labeling systems as a control, cross-reactivities were excluded. The sections were mounted with Kaiser's glycerol gelatine (Merck, Vienna, Austria), microphotography was taken using an Axiophot microscope (Zeiss, Oberkochen, Germany).

### Image segmentation and quantitative analysis

The digital color images were segmented in order to get binarized representations of the distinct and individual cell areas. These segmented images were used to analyze the spatial distribution of the cells. Particularly, the segmented cell areas yielded a quantitative measure, which could be interpreted as a quantitative description of cell migration.

At first, the area of the vessel lumen had to be eliminated interactively because this tissue should not be included in the analysis. The segmentation task was subdivided into three sub parts, the total specimen, the red-stained CD56^+^ cells or CXCR3^+^ cells and the blue-stained areas of the endothelium or of the muscular layer of spiral arteries. For every sub-part the digital color images (Figure 1 gives examples) served as bases of operations.

### Total specimen

**Figure 3:**
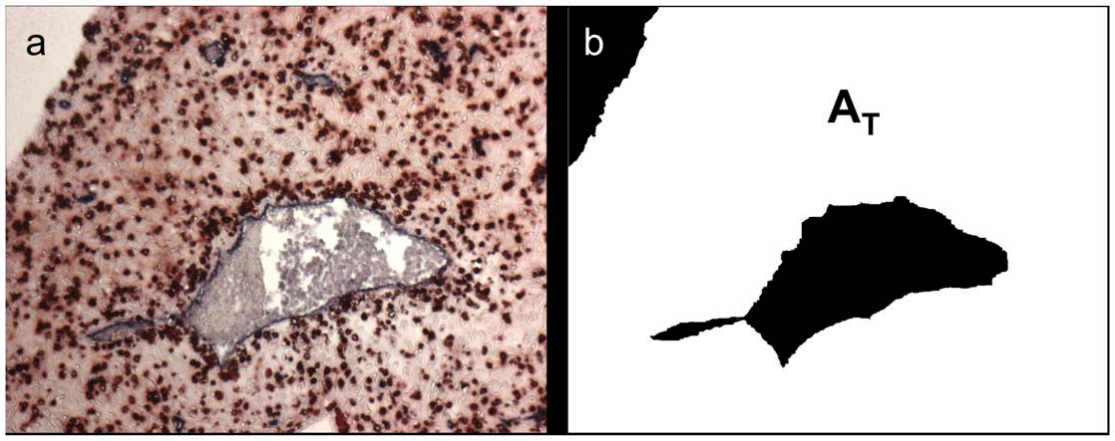
Binarized image using Region of Interest defined by interactive drawing. (a) Source image. (b) The binary (white) area is representing the total specimen's area A_T_.

In order to eliminate the background, the specimen as a whole was segmented by an interactive drawing of a region of interest (ROI). This ROI excluded areas of the microphotography outside the tissue section, inside larger vessels and also tissue areas the inclusion of which would skew the analysis (such as glands or, for analysis of vessels without a muscular layer, spiral arteries). The background as well as the interior of very large vessels (which could not be detected by means of automatic image segmentation) were filled with black pixels. An example is shown in Figure 3b. The resulting binary (black and white) modality represented the specimen's area. The total number of white pixels gave the total area of the specimen *A_T_*. This area served as a mask for the following image processing steps.

### Analysis of mobile cells

**Figure 4:**
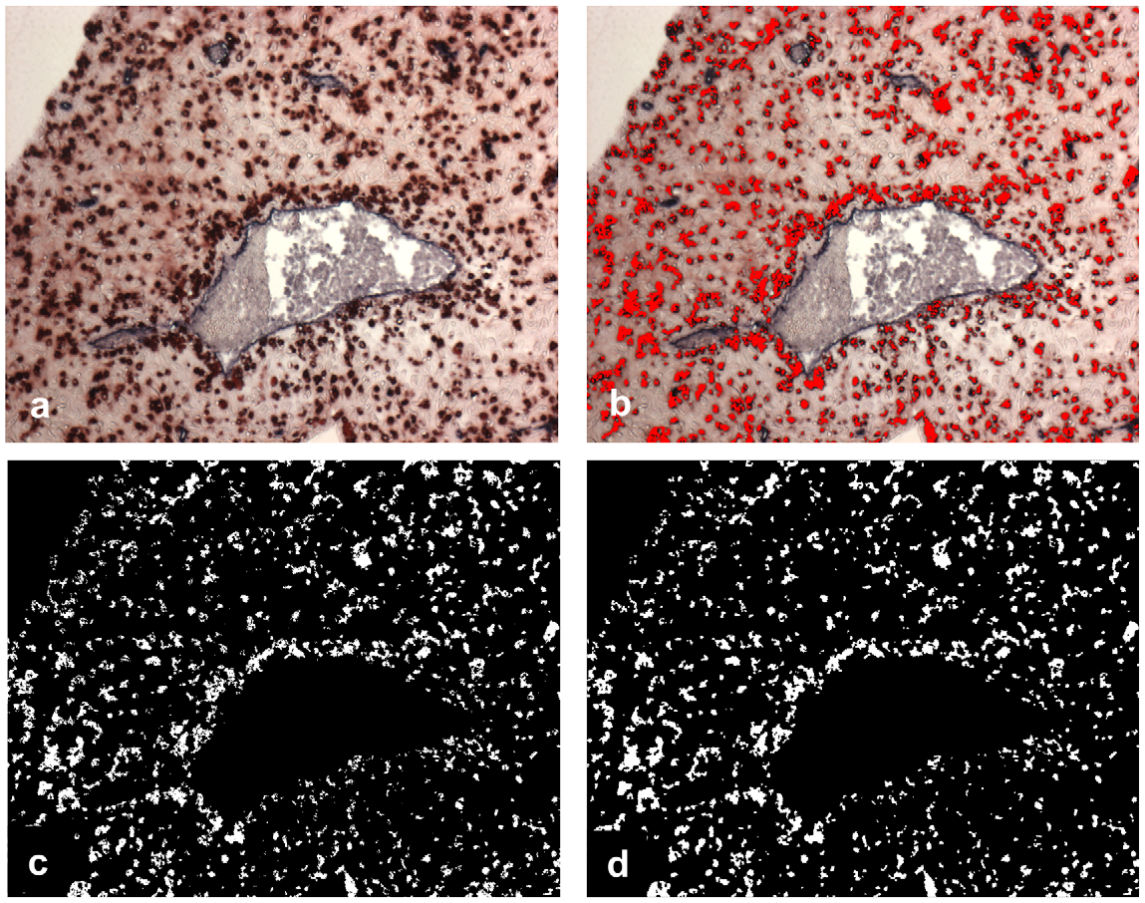
Segmentation of the mobile cells. (In this example: CD56^+^ cells). The color image (a) is segmented by relative RGB color planes (b, c). Some morphological operations (closing, opening) yield the red stained area A_R_ (d).

The red stained cells in the photograph (CD56^+^ cells or CXCR3^+^ cells) were segmented using relative grey values of the RGB color planes of the original color image. All pixels which had a grey value in the red color plane, that was at least 60% higher than the grey values of the green or the blue color planes, were set to white, all other pixels were set to black (Figure 4c). For clarification, Figure 4b depicts an overlay of these white pixels onto the original color image, where all the segmented white pixels were set to an artificial red color. The use of these relative grey values was appropriate and very stable resolving staining and intensity variations very well. Some morphological operations (closing and opening) were applied in order to eliminate residual noise and granular textures (Figure 4d). This last image represented the segmented and binarized (black and white) red stained cells and the total number of white pixels in Figure 4d gave the red-stained cell area *A_R_*.

### Analysis of fixed tissue structures

**Figure 5:**
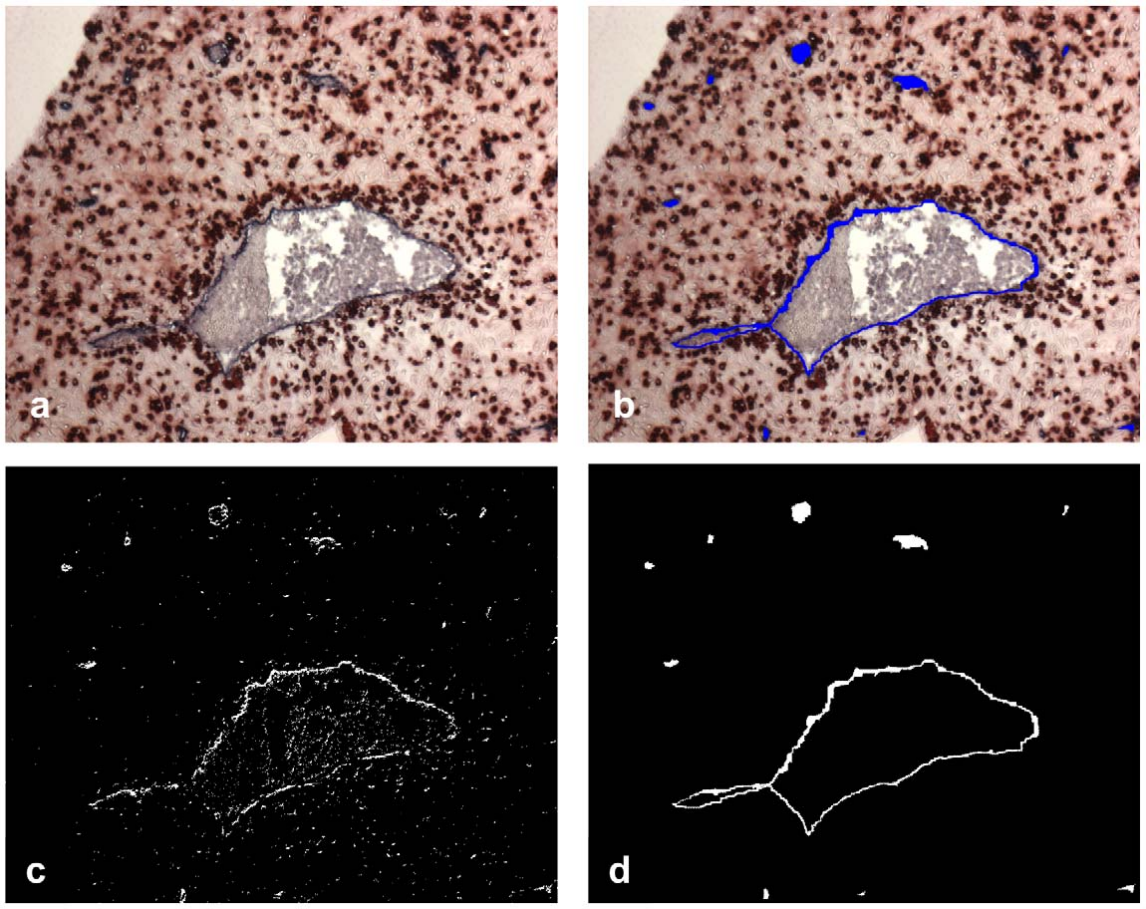
Segmentation of the fixed structure. The color image (a) is segmented by relative RGB color planes (b, c). Some morphological operations (scrapping of small objects, closing) yield the blue stained area A_B_ (d).

The fixed tissue structures in the photograph (blue stained endothelium or muscular layer of vessels) was segmented and binarized with the relative grey values of the RGB color planes as well. Similarly some morphological operations were applied. In this case these operations included a scrapping of all objects less than 0.01% followed by a moderate closing with a 11x11 kernel. The subsequential image processing steps can be seen in Figure 5. Accordingly, the total number of white pixels in Figure 5d represents the segmented and binarized blue stained endothelial area *A_B_*.

### Quantitative migration analysis

In order to elaborate a local distribution measure of the mobile cells, it was necessary to define certain neighborhood areas around their fixed tissue structures. These neighborhood areas allowed us the calculation of cell number fractions, serving as a quantitative migration measure.

Vessel neighborhood areas were gained by morphological dilation of the already segmented and binarized vessel images.

If 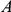 and 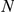 are elements of a two dimensional image space 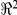, the dilation is defined as:
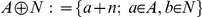



where 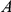 is the set of object pixels, 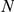 is a dilation kernel and 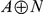 is the dilated set 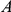.

Several dilation kernels with increasing sizes were used in order to define neighborhood areas of increasing width. Actually, kernel matrices starting with 31 pixels x 31 pixels and stepwise increased with 30 pixels were used as dilation kernels. With a scale bar of 100 µm = 150 pixels the series of dilation kernels 31×31, 61×61, 91×91, etc. in pixel units in the image space corresponded to a neighborhood width of 10, 20, 30 µm etc. in the real object space.

**Figure 6:**
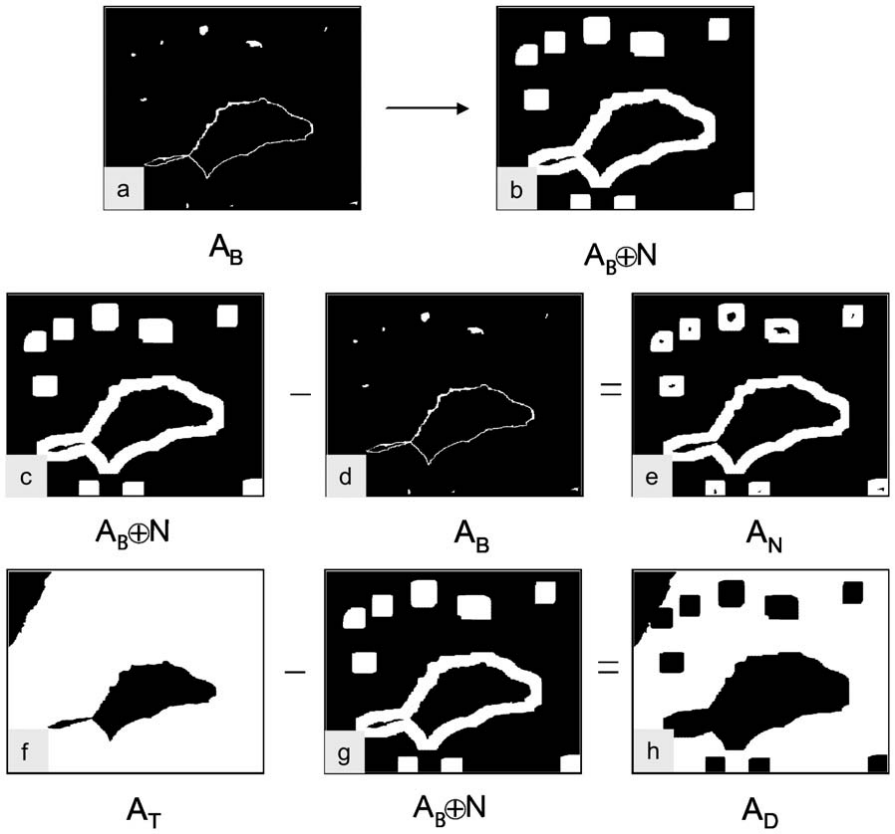
Definition of the endothelial neighborhood by morphological dilation. The segmented image of the endothelium (a) is morphologically dilated (b). (Here a neighborhood width of 30 µm is chosen.) The dilated image A_B_ + N (c) subtracted with the blue stained endothelial area A_B_ (d) yields the endothelial neighborhood area A_N_ (e). The total specimen area A_T_ (f) subtracted by the dilated area A_B_ + N (g) yields the residual decidua area A_D_ (h). A_N_: Neighborhood area, A_D_: Area of residual decidua, A_B_: Area of endothelium, A_T_: Area of total specimen.

Figure 6a depicts an example of an endothelium area 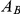 and Figure 6b shows an example of the dilated area 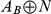 using a 20 µm distance (61×61) dilation kernel. The actual neighborhood area 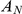 was gained by subtracting the endothelium area 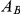 from the dilated area 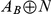.
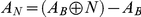



Additionally, for every distinct size of dilation kernel the residual decidua area 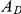 was calculated by subtracting the dilated areas 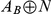 from the total specimen areas 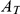.
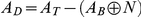



**Figure 7:**
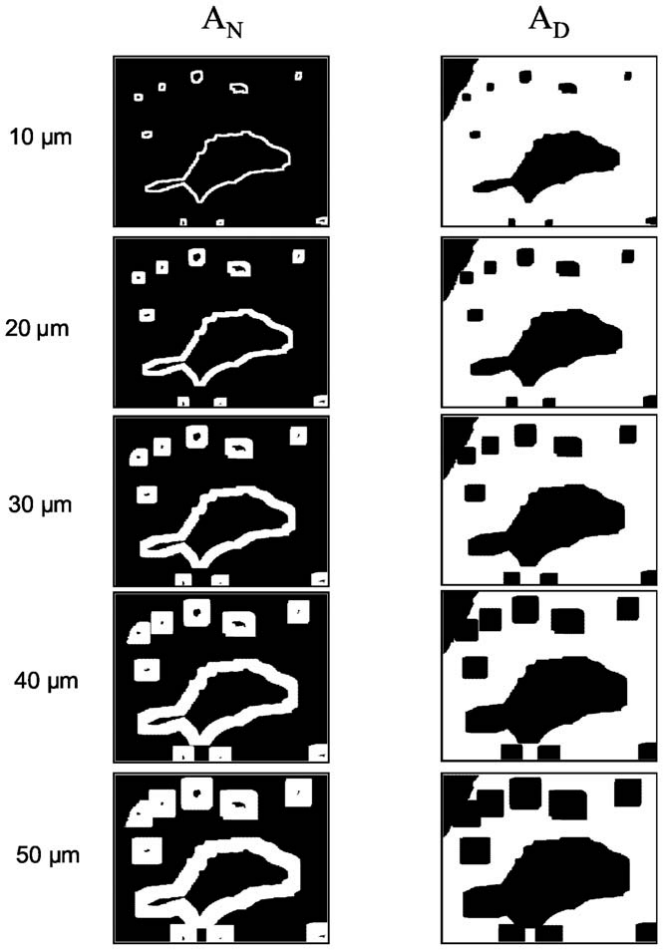
Endothelial neighborhood (A_N_) and area of residual decidua (A_D_) with increasing neighborhood width.

Examples of the calculations of the areas 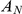 and 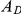 are depicted in Figure 6c–h. Figure 7 shows these areas 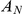 and 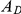 from the top to the bottom for neighborhood widths of 10 µm up to 50 µm. Accordingly, with increasing dilation kernel size, the neighborhood areas increase and the residual decidua areas decrease.

Finally the areas of the red stained CD56^+^ or CXCR3^+^ cells located within a distinct vessel neighborhood 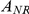 or located within a residual decidua area 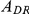 were calculated by a Boolean AND combination of the red stained cell areas 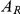 with the neighborhood areas 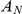 or the residual deciduas areas 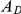:
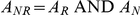


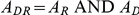



**Figure 8:**
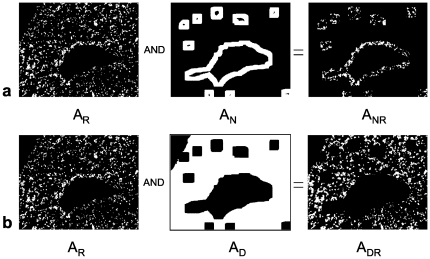
Boolean operations. (a) Definition of the area of red stained cells located within the endothelial neighborhood (A_NR_). (b) Area of red stained cells located within the residual decidua (A_DR_).

Figure 8a shows example images for 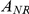 and Figure 8b for 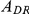.

Finally, normalized fractions of these areas were calculated for the neighborhood and for the decidua areas as well.
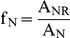


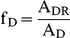



where 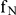 is the normalized fraction of red stained cells located in the vessel neighborhood and 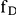 is the normalized fraction of red stained cells in the corresponding residual deciduas.

These fractions give quantitative values (in percentages) of mobile cell densities (in our examples CD56^+^ or CXCR3^+^ cells located in the neighborhood of fixed tissue structures (in our examples vascular endothelium or the muscular layer of vessels) or located in the residual decidua. The fraction 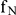 as well as the fraction 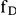 has the unit 1 and therefore it is not necessary to calculate the distinct areas 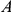 in real space units (µm). As a result, the actual values of these areas were obtained by calculating the number of the corresponding white pixels in the image space. The image processing and the quantitative analysis were performed with IDL (Interactive Data Language, ITT Visual Information Solutions, Boulder, USA).

### Simulation of randomly distributed cells

**Figure 9:**
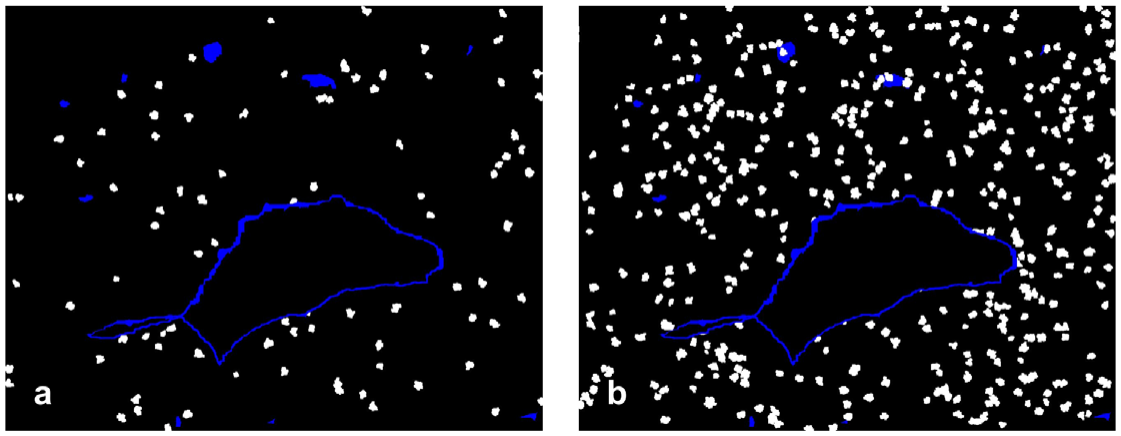
Artificial images with randomly scattered cell distributions. For the sake of better comparison of cell distributions at different overall cell densities a model of random distribution of artificial cells in relation to neighborhood areas and rest decidua of a specific sample was generated. Randomly distributed cells projected onto a sample object of the total specimen's area A_T_ (see Figure 3b). (a) Low cell density with 100 seeds, (b) high cell density with 500 seeds.

The verification of the morphometric analysis was performed by generating artificial digital images of randomly spreaded cells. Completely black images without any objects, but with identical width and height were created. An initial number of seed points was specified, particularly 100, 200, 300, 400 and 500 for this simulation. The number of seed points defined the cell density in an image. These seed points were spatially distributed abroad the image using a digital random generator. Each seed point appeared as a white pixel in the image and reflected a seed point of a single cell. Then, these seed points were enlarged by adding white pixels at random neighboring locations. The enlargement process was repeated until the growing artificial cells had a size that matched the size of the real cells. The artificial cells showed a strong morphometric similarity to the real cells, mainly because of the random enlargement process. A sample image of a segmented vessel was taken and some overlay images were created. Cells inside the vessel and outside of the specimen were eliminated, thus were restricted to the total area 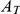. Figure 9 illustrates these artificial images with two different numbers of seed points. For every number of initial seeds a number of 100 images was generated, yielding 500 distinct images altogether. Each image was different, because of the random number of seeds and the random enlargement process.

### Statistical analysis

Because of unknown non-normal distributional features, nonparametric statistical techniques were used throughout. For determination of the significance of differences in distribution densities the paired Wilcoxon test was applied (level of significance 0.01). Two-tailed p-values were determined. In cases where more than one tissue sample was available from a single patient, information was summarized by the median of cell densities of the samples from this patient. Hence, testing was based on one robust observation per each patient. Two-tailed P values were determined. Calculations were done using the SPSS 16 software.

## Results

### Distribution of CD56^+^ cells and of CXCR3^+^ cells in the decidua of first trimester pregnancy in relation to endothelium of vessels devoid of a layer of smooth vasculature

**Figure 10:**
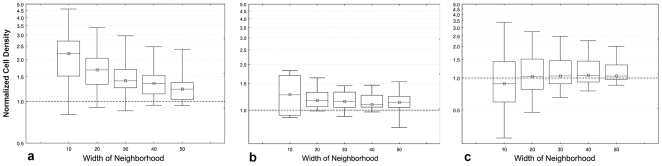
Results of analysis of samples. (a) Relative density of CXCR3^+^ cells in neighborhood of capillaries and venules. (b) Relative density of CD56^+^ cells in neighborhood of capillaries and venules. (c) Relative density of CD56^+^ cells in neighborhood of spiral arteries. The data are individually normalized in relation to density in residual decidua = 1. (y axis, log scale.)

**Figure 11:**
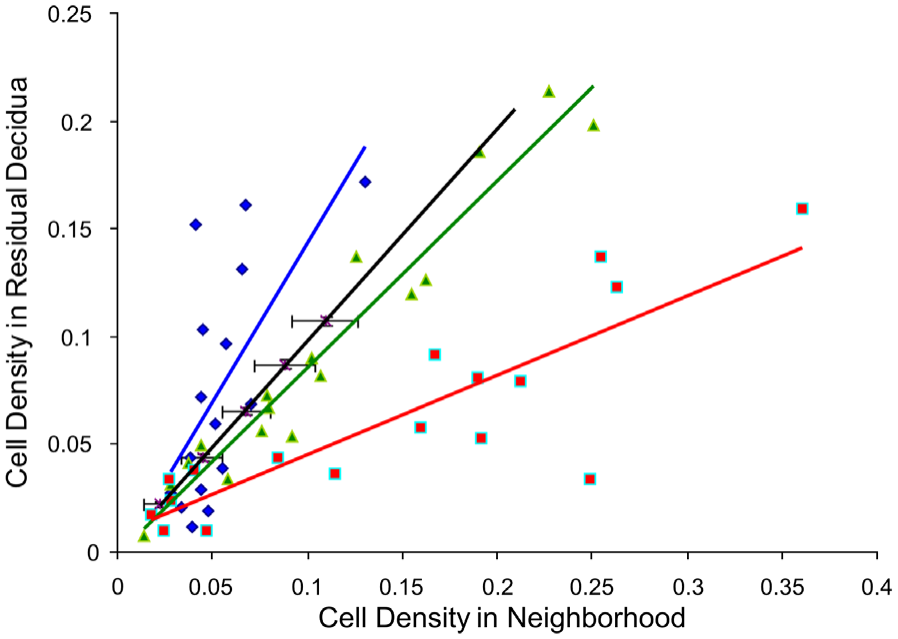
Quantile - quantile plot for neighborhood 10 µm. The dots (red: CXCR3^+^ cells in relation to endothelium, green: CD56^+^ cells in relation to endothelium, blue: CD56^+^ cells in relation to the tunica media of spiral arteries) represent the medians of patients' data of cell densities in the neighborhood (x-axis) versus in the residual decidua (y-axis). The black line represents the random distribution, the black bars the standard deviation of the random distribution.

For all widths of the neighborhood areas tested (from the smallest size of 0–10 µm up to a width of 0–50 µm around the vessel endothelium, in increments of 10 µm) a highly significant accumulation of CXCR3^+^ cells was found in the vicinity of endothelium of vessels which do not contain a muscular layer, as compared to the cell density in the residual decidua (Figure 10a; p<0.001 for all tested neighborhood widths). Also for CD56^+^ cells we found accumulation in the vicinity of this type of vessels (Figure 10b). (Significance in relation to respective neighborhood widths: 10 µm: p<0.005; 20 µm: p<0.002; 30 µm: p<0.011; 40 µm: p<0.002; 50 µm: p<0.022.) The quantile-quantile plot reflects this tendency of periendothelial accumulation in comparison to a random distribution (Figure 11).

### Distribution of CD56+ cells in relation to vascular smooth vasculature

No significant difference in the density of CD56^+^ cells was found in the neighborhood of vessels containing a layer of smooth muscle cells as compared to the areas of decidua distant from these vessels (Figure 10c, Figure 11).

## Discussion

We have developed a method for automated morphometric analysis of the relative frequency of specific single cells in tissue sections in the neighborhood of fixed structures, based on 2-color staining. This method should be able to replace labor-intensive methods of visual enumeration not only because it is less labor-consuming but also because of the objectivity of the analysis [13]. We anticipate applicability of the method for a variety of other studies, such as e.g. for accumulation of specific single cells in the vicinity of tumors, of spheroids in 3-dimensional confrontation culture models, of invasive trophoblast in the placenta, of other epithelial structures, or of implanted artificial materials. Following injection of pre-labeled cells into the blood distribution of these cells in tissues might be studied in *ex vivo* models.

In our examples of applications, we have objectified an accumulation of CD56^+^ cells in the neighborhood of endothelium of venules and capillaries of first trimester decidua. It has been reported from mouse pregnancy that 25–35% of uNK cells in decidua basalis and 15–20% of uNK cells in the mesometrial lymphoid aggregate at d 10–12 of pregnancy are perivascular [13]. This meant that they were positioned in the vascular smooth muscle layer of the larger vessels or as the cell next to the endothelium in smaller vessels. However, it may well be that the vessel-associated group was underestimated as uNK cells may promote the development of new sprouting vessels that would be tiny.

Regarding perivascular accumulation of CXCR3^+^ cells which (while not being a perfectly homogeneous cell type) are mostly uNK cells, our data are compatible with the concept that this receptor is relevant for recruitment of peripheral blood NK cells to the decidua.

It is well established that uNK cell derived IFNγ plays a significant role in vascular remodeling of spiral arteries [11], [14]. Earlier it has been described that in non-pregnant endometrium periarterial and periglandular accumulation of NK cells takes place during early secretory phase which then gives place to dense scattering of these cells throughout the stroma [15]. Our data do not show uNK cells accumulating around unremodeled spiral arteries of first trimester parietal decidua, which is in agreement with recently published results showing that accumulation of leukocyte around this vessels coincides with remodeling, i. e., loss of vascular smooth muscle cells [16].
